# Statistical reporting inconsistencies in experimental philosophy

**DOI:** 10.1371/journal.pone.0194360

**Published:** 2018-04-12

**Authors:** Matteo Colombo, Georgi Duev, Michèle B. Nuijten, Jan Sprenger

**Affiliations:** 1 Department of Philosophy and Tilburg Center for Logic, Ethics and Philosophy of Science (TiLPS), Tilburg University, Tilburg, The Netherlands; 2 Center for Economic Research (CentER), Tilburg School of Economics and Management, Tiilburg University, Tilburg, The Netherlands; 3 Department of Methodology and Statistics, Tilburg University, Tilburg, The Netherlands; 4 Department of Philosophy and Educational Sciences and Center for Logic, Language and Cognition (LLC), Università degli Studi di Torino, Turin, Italy; Coventry University, UNITED KINGDOM

## Abstract

Experimental philosophy (x-phi) is a young field of research in the intersection of philosophy and psychology. It aims to make progress on philosophical questions by using experimental methods traditionally associated with the psychological and behavioral sciences, such as null hypothesis significance testing (NHST). Motivated by recent discussions about a methodological crisis in the behavioral sciences, questions have been raised about the methodological standards of x-phi. Here, we focus on one aspect of this question, namely the *rate of inconsistencies in statistical reporting*. Previous research has examined the extent to which published articles in psychology and other behavioral sciences present statistical inconsistencies in reporting the results of NHST. In this study, we used the R package statcheck to detect statistical inconsistencies in x-phi, and compared rates of inconsistencies in psychology and philosophy. We found that rates of inconsistencies in x-phi are lower than in the psychological and behavioral sciences. From the point of view of statistical reporting consistency, x-phi seems to do no worse, and perhaps even better, than psychological science.

## Introduction

Experimental philosophy (x-phi) is a young field at the intersection of philosophy and psychology that aims to make progress on philosophical questions by using experimental methods traditionally associated with the psychological and behavioral sciences [1–2].

Those sciences are, however, undergoing a methodological crisis regarding the reproducibility and statistical correctness of experimental research [3–6];. This raises the question of whether x-phi is equally affected by this crisis, or whether notable differences can be found.

One aspect of the methodological crisis in psychology is the high rate of reporting inconsistencies in the statistical analysis of data; roughly half of the published papers in psychology contain at least one *inconsistent result* where the reported *p*-value does not match the reported value and degrees of freedom of the test statistic. Around one in eight papers contain at least one *gross inconsistency*, in which the reported *p*-value is significant, but the recalculated *p*-value based on the reported degrees of freedom and test statistic is not, or vice versa [7].

Regardless of whether or not such inconsistencies are due to honest mistakes or to questionable research practices, they can have serious consequences. They can give rise to ill-founded arguments and erroneous conclusions about the reality of observed effects; they can bias meta-analyses and effect size estimates [8]; and they can affect the reputation of an entire discipline. In sum, consistent statistical reporting is a necessary (but by no means sufficient) characteristic of a methodologically healthy scientific discipline.

Data on the rate of statistical inconsistencies are not available for x-phi. Making these data available is important for a variety of disciplines, such as philosophy, psychology, and linguistics. Results from x-phi have been used to object to an exclusive reliance on intuition as a source of justification for philosophical arguments, but also as relevant psychological evidence concerning central concepts such as knowledge and belief [9–10], intentional action [11–12], the meaning of proper names [13–14], freedom and determinism [15–16], consciousness [17–18], and causal and moral responsibility [19–20].

Similar to psychologists and behavioral scientists, experimental philosophers typically analyze their data with null hypothesis significance testing (NHST). It is thus not implausible to hypothesize that the rates of reporting inconsistencies will be similar. At the same time, x-phi differs from experimental psychology in relevant respects: first, it is a genuinely interdisciplinary field often involving collaboration between researchers with different backgrounds; second, researchers in x-phi are mostly trained as philosophers and have rarely received a formal training in statistics.

In this study, we evaluated this hypothesis on a sample of 220 x-phi articles from the *PhilPapers* database, using the R package *statcheck* [3] that automatically extracts statistical results and recalculates *p*-values. We also compared different subfields of x-phi taken from the conventional categorization in PhilPapers, which correspond to different types of philosophical questions submitted to experimental testing. Finally, we evaluated trends of (gross) inconsistencies over time, and contrasted our findings with the findings of previous studies on the prevalence of reporting inconsistencies in psychology, and other disciplines in the social and behavioral sciences.

## Material and methods

### Sample

**Fig 1:**
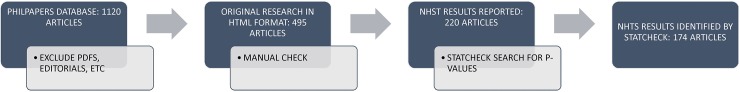
A schematic representation of our sampling procedure.

The articles in our sample and their topic classification were extracted from PhilPapers.org, the largest search index and bibliography of philosophical research. Our initial sample consisted of 1,120 papers in PhilPapers classified as “Experimental Philosophy” as of September 6, 2016 (https://philpapers.org/browse/experimental-philosophy). Any paper added later was not included in our sample. Published papers came from over 150 journals, the great majority of which were philosophy journals. We excluded editorials and commentaries because they did not contain original research, and all PhD dissertations because they often overlapped with journal articles. We also excluded all articles that were only available in PDF format since statcheck can have trouble to process PDF files reliably [7]. This left us with 495 unique articles available in HTML format, consisting of journal articles, book chapters, and working papers deposited in professional online archives. We then conducted a manual check and eliminated all articles that did not contain NHST results. The final sample on which statcheck was run contained 220 articles. See Fig 1 for a schematic representation of the sampling procedure.

Articles in PhilPapers are categorized using a mixture of automatic tools and user contributions. For instance, all articles that appear in journals associated with a certain area (e.g., *The British Journal for the Philosophy of Science*) are automatically categorized in that area (in this case, philosophy of science). Following the PhilPapers categorization system, we organized the papers in our sample into eight subfields: Action, Ethics, Epistemology, Language, Mind, Metaphysics, Foundations of experimental philosophy, and Miscellaneous. Multiple classifications were possible, and the same paper can be classified in more than one category. For details about the categorization system of PhilPapers, see https://philpapers.org/help/categorization.html.

### “statcheck”

We used the R package statcheck developed by one of the authors (M.N.) in order to check for statistical reporting inconsistencies in the articles [3]. The statcheck package (see also http://statcheck.io) converts a pdf or html file into a plain text file from which it extracts the *t*, *F*, *r*, *χ*^*2*^ and *z* statistics, with the accompanying degrees of freedom (*df*) and *p*-values. Statcheck then recalculates the *p*-value with the reported test statistic and *df* and compares it to the value reported in the article. When these two values differ by more than the allowed tolerance margin (e.g., due to rounding), statcheck reports an *inconsistency*. If the discrepancy changes the statistical conclusion from non-significant to significant or vice versa, statcheck reports a *gross inconsistency*. Statcheck can only detect results that are reported completely and according to the APA guidelines. Statcheck takes into account one-sided testing: if a *p*-value would have been consistent if the test was one-sided, *and* somewhere in the full text of the article statcheck detected the words “one-tailed”, “one-sided”, or “directional”, the result is counted as consistent. While the focus on HTML articles and APA style reporting may introduce a non-random component, we do not see how it would affect our expectations on rates of reported inconsistencies.

The overall accuracy of statcheck in flagging (gross) inconsistencies ranges from 96.2% to 99.9%, depending on specific settings [21]. Statcheck cannot indicate which of the three components of a result caused an inconsistency (test statistic, degrees of freedom, or *p*-value), and it cannot say anything about whether an inconsistency was an innocent typo, an intentional error, or anything in between. The tool is best compared to a spell check for statistics.

We performed the analysis on a Mac OS because for our set of articles, statcheck was most successful at extracting results using this operating system. Since experimental philosophy is still a young discipline, and since no similar research has been conducted in the past, we refrained from testing a precise hypothesis on the rate of inconsistencies and used descriptive statistics only. The results of our study, however, motivate hypotheses that can be tested in future research. All data are available in the Open Science Framework repository at the URL https://osf.io/rg5p4/.

## Results

### Prevalence of NHST results

Statcheck detected 2,573 NHST results, distributed over 174 out of 220 files in our final sample (79.1%). The percentage of articles with NHST results is high for all but one of the subfields of experimental philosophy (64.0–85.3%; see Table 1 for details) and much higher than what Nuijten et al. [7] found in their sample of psychology journals (54.4%). The only exception was for “Foundations of Experimental Philosophy”, where 38.5% of the articles contained NHST results. The divergence can be explained by the fact that we conducted a manual check for the presence of NHST results before including an article in our final sample.

**Table 1:** Summary statistics for our sample and the relevant subfields. Specifications of the years from which HTML articles were available, the number of articles in our sample, the number of articles with NHST results reported in APA style, the number of NHST results, and the median number of APA reported NHST results per article. Articles could be classified into multiple subfields.

Field	Years included	# Articles in final sample (after manual check for NHST results)	# Articles with NHST results in APA format	# NHST results	Median # NHST results per article with NHST results
Total (without duplicates)	1993–2016	220	174 (79.1%)	2,573	11.0
Action	2005–2016	75	64 (85.3%)	1122	12.0
Ethics	1993–2016	53	34 (64.2%)	641	16.0
Epistemology	2007–2015	25	21 (84.0%)	364	10.0
Language	2009–2015	25	17 (68.0%)	283	14.0
Mind	2009–2016	25	16 (64.0%)	191	10.5
Metaphysics	2007–2016	18	13 (72.2%)	156	7.0
Foundations of Experimental Philosophy	2010–2015	13	5 (38.5%)	56	11.0
Miscellaneous	2006–2015	12	8 (66.7%)	135	10.0

### (Gross) inconsistencies: General prevalence

In total, statcheck recognized NHST results in 174 articles in our final sample of 220 papers. This means that for the remaining 46 papers, results were either reported incompletely or not in APA style. We calculated the average proportion of inconsistent NHST results per article, and also the rates of articles that contained inconsistent results. These numbers were also split up per subfield and over time. One outlier that contained lots of inconsistent results, was removed manually in this analysis. The article in question was Wright, J.C., & Bengson, J. (2009). Asymmetries in Judgments of Responsibility and Intentional Action, *Mind and Language*, 24(1), 24–50. We detected 15 *χ*^*2*^-tests in this article that were all inconsistencies, nine of which were gross inconsistencies. In all cases, the reported and recalculated *p*-values were very far apart (e.g., *χ*^*2*^ (120) = 9.8, *p* = .002; recomputed *p* = 1). We speculated that the authors reported the wrong degrees of freedom, the wrong tail of the distribution, or that something else went wrong. We decided to treat this article as an outlier and exclude it from our analyses to not inflate our estimates of the general prevalence of statistical reporting inconsistencies. This means that inconsistency rates were calculated relative to 173 articles and 2,558 NHST results.

Across all journals and years, 67 out of 173 articles (38.73%) showed at least one inconsistency in statistical reporting. Of all 2,558 NHST results/p-values that were reported, 160 were inconsistent (6.25%). See Table 2 for details. This means that on average, 1 out of 16 reported p-values is inconsistent. These percentages are a bit lower than what has been found in psychology with 49.6% (8,273/16,695) of articles showing at least one inconsistency and 9.7% of inconsistent p-values.

**Table 2:** General prevalence of inconsistencies for the articles in the current study, relative to those articles that contained NHST results (N = 173).

Category	Absolute Number	Percentage
Articles with at least one inconsistency	67	38.73%
Articles with at least one gross inconsistency	11	6.36%
P-values that are inconsistent	160	6.25%
P-values that are grossly inconsistent	13	0.51%
Average % of p-values per article that is inconsistent*	-	6.85%
Average % of p-values per article that is grossly inconsistent*	-	0.41%

* this percentage takes dependency of p-value within an article into account.

Similarly, 11 out of 173 articles (6.36%) that reported NHST results showed at least one gross inconsistency in statistical reporting, i.e., the recalculation changed the result from non-significant to significant or vice versa. This is again lower than in psychology where 12.9% of all articles contained at least one gross inconsistency. Of all 2,558 NHST results/p-values that were reported, 13 were grossly inconsistent (0.51%). We conducted a manual check of the involved studies and observed that the grossly inconsistent p-values concerned central hypotheses of the study.

### (Gross) inconsistencies: Prevalence and per subfield

**Fig 2:**
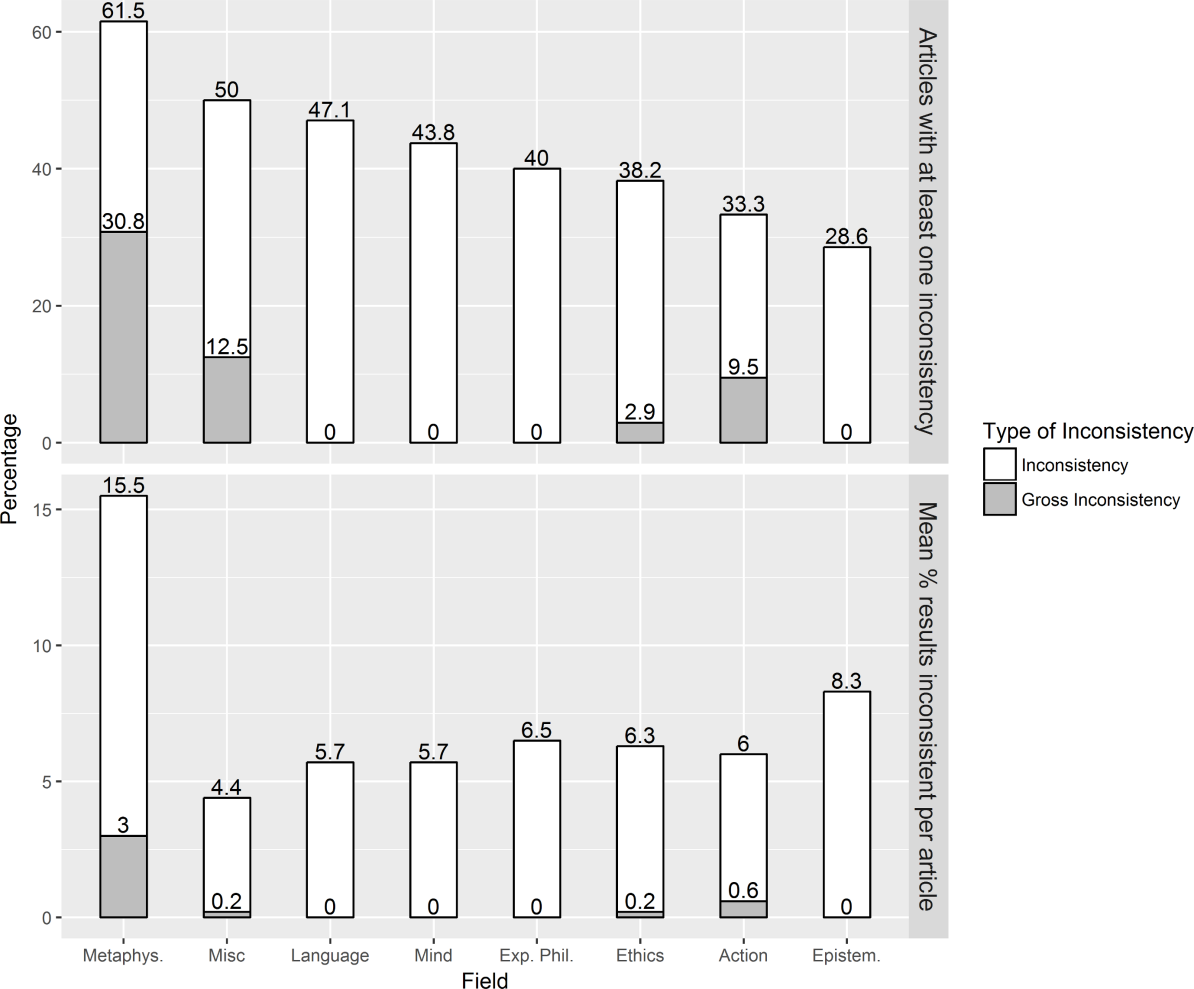
The average percentage of articles within a field with at least one (gross) inconsistency and the average percentage of (grossly) inconsistent p-values per article, split up by field. Inconsistencies are depicted in white and gross inconsistencies in grey. For the fields Action, Epistemology, Ethics, Foundations of Exp. Phil., Language, Metaphysics, Mind, and Misc, respectively, the number of articles with null-hypothesis significance testing (NHST) results is 63, 21, 34, 5, 17, 13, 16, 8, and the average number of NHST results in an article is 17.1, 17.3, 18.9, 11.2, 16.6, 12.0, 11.9, and 16.9, for the fields respectively.

The white bars in Fig 2 display how reporting inconsistencies are distributed across the different subfields of x-phi. This analysis is primarily of interest to philosophers, who may want to understand how and why rates of reporting inconsistencies differ in different subfields of the discipline.

We found that roughly every second article in Metaphysics, Miscellaneous and Philosophy of Language contains an inconsistency, but these rates are substantially lower in the other subfields (especially Epistemology: 28.6%). When one looks at the distributions of gross reporting inconsistencies per subfield; 30.8% of all articles in Metaphysics, 12.5% of all articles in Miscellaneous and 9.5% of articles in Philosophy of Action report at least one grossly inconsistent p-value. All other domains have zero or negligible rates of articles that report grossly inconsistent results.

While interesting, however, these findings are to be treated with caution because the number of NHST results in an article varies considerably per subfield, and also because the articles included in our final sample are not necessarily representative of the overall population per subfield.

Notably, the percentage of non-significant results reported as significant, relative to all NHST results, was higher than the percentage of significant results reported as non-significant (0.56% vs. 0.39%), replicating a similar finding from psychology (1.56% vs. 0.97%; [7]), although the effect was smaller for x-phi and the sample size too small to draw reliable inferences.

### (Gross) inconsistencies: Developments over time

In recent years, social and behavioral scientists have paid close attention to questionable research practices (QRPs), such as HARKing and p-hacking [22]. Statistical inconsistencies, such as reporting non-significant p-values as significant, can be taken as an indicator of the prevalence of such practices [5]. We have therefore looked at the rate of (grossly) inconsistent results over time in order to obtain a very rough indication of whether the prevalence of QRPs has been increasing or decreasing in x-phi.

**Fig 3:**
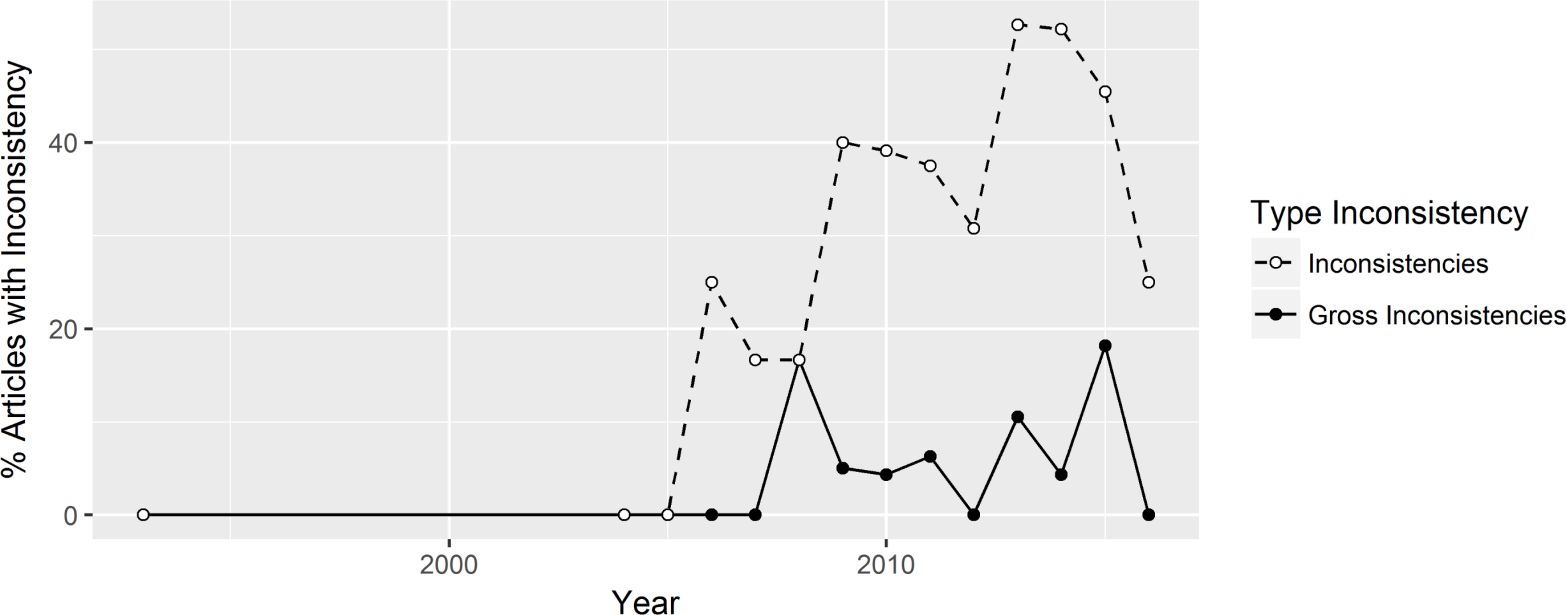
Percentage of articles with at least one inconsistency (open circles) or at least one gross inconsistency (solid circles) over time.

**Fig 4:**
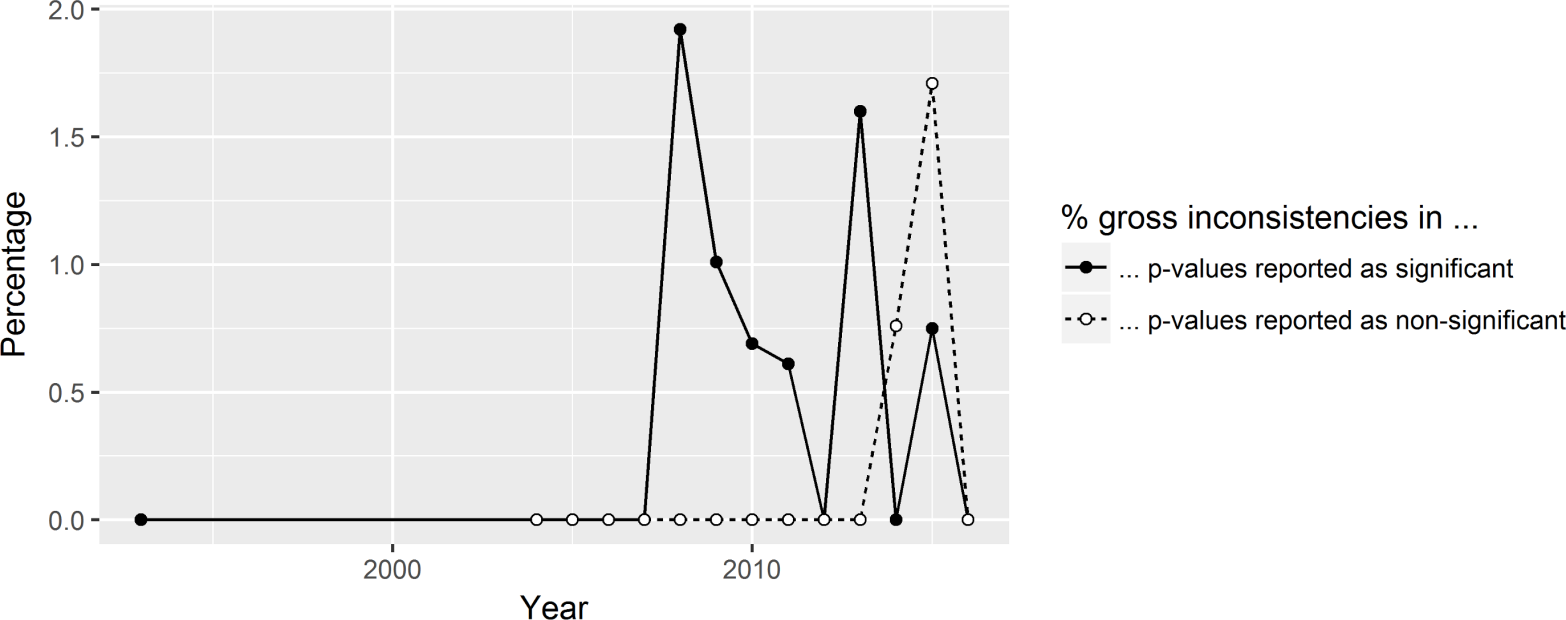
The percentage of gross inconsistencies in p-values reported as significant (solid line) and nonsignificant (dotted line), over the years.

Fig 3 shows the percentage of (grossly) inconsistent p-values over time. The number of statistical inconsistencies has been rising steadily until 2013. Since then, it has been falling again. A possible explanation is that recent debates on statistical methods have led to more attention to statistical reporting. For gross inconsistencies, there is no obvious trend. The same can be said about the way gross inconsistencies are split up into p-values erroneously reported as significant and non-significant, respectively (Fig 4). Thus, these results cannot provide us with any indication about the prevalence of QRPs in x-phi.

**Fig 5:**
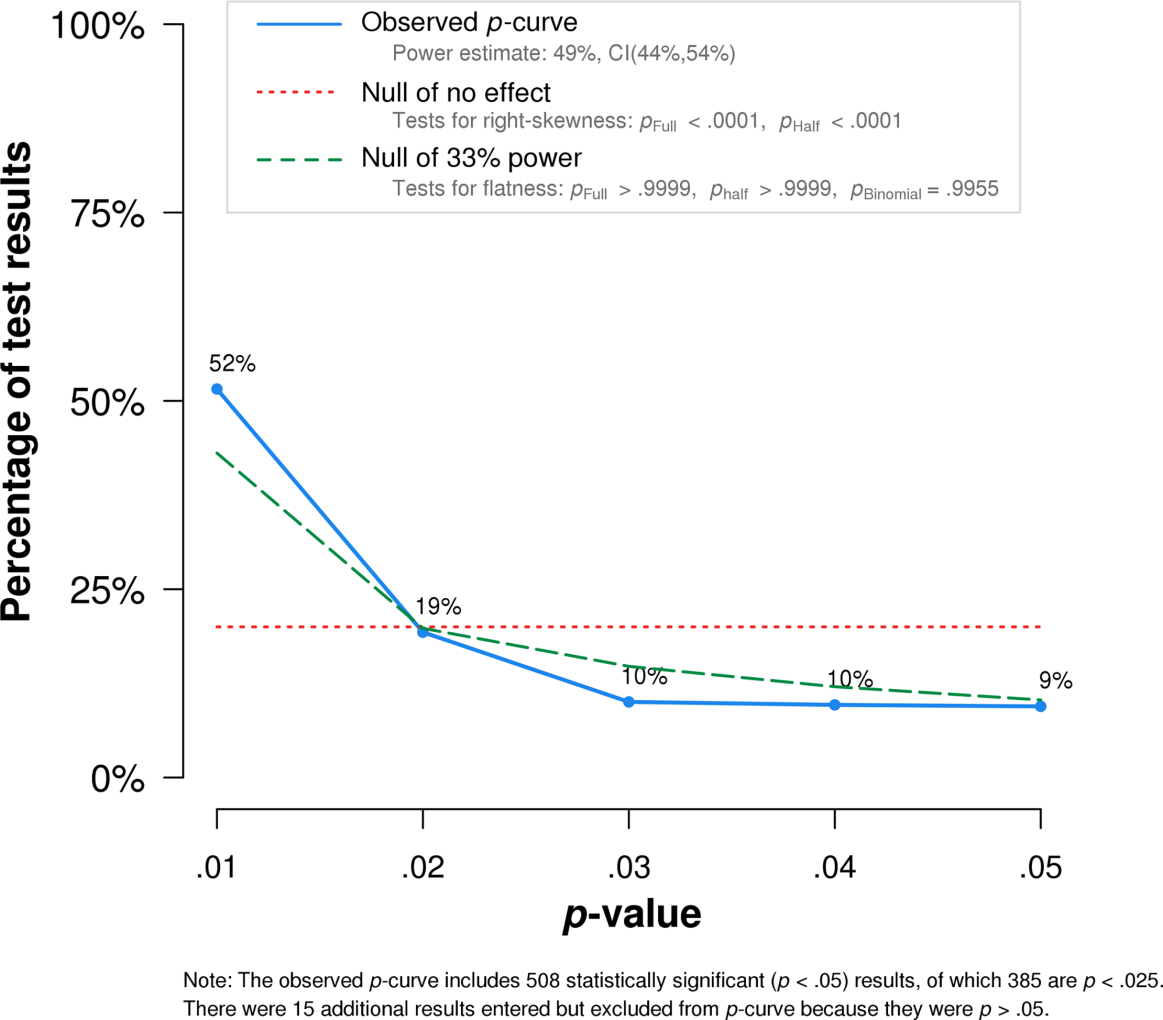
A p-curve analysis for the reported p-values in our final sample. The actual distribution of p-values is compared to the distribution expected under a true null hypothesis and a hypothesis of 33% power. Note that 20 results were automatically excluded from the *p*-curve because they were not < .05 upon recalculation.

Finally, Fig 5 presents a *p*-curve analysis that uses the distribution of significant *p*-values to quantify the evidential value of a set of results [23–24]. This analysis is based on the notion that if the *p*-values are in fact false positives (there is no effect in the population), their distribution would be uniform. Conversely, if there is an effect in the population, the *p*-value distribution would be strongly right-skewed. Here, the *p*-curve is clearly right-skewed, which indicates the presence of evidential value. This finding is in line with the generally favorable findings of the x-phi replication project by Cova et al. [25]. Similarly, the test for flatness (against the 33% power null hypothesis) does not indicate that the effects in our dataset are too small for the given sample sizes and that new studies should use better powered samples. That said, the results of the *p*-curve analysis have to be interpreted with care because the set of studies and *p*-values is highly heterogeneous. The analysis is based on the reported *p*-values; it does not change when performed on the *p*-values recalculated by statcheck on the basis of the test statistics.

### Comparison to other fields of social science

Table 3 compares the results of this study to analogous in psychology conducted in the last years. X-phi reports fewer inconsistencies than psychological science, both in terms of the percentage of articles that report a (gross) inconsistency, and in terms of the overall percentage of (grossly) inconsistent NHST results. The fact that x-phi has lower inconsistency rates than what all five benchmark studies in psychological science report, sometimes with considerable effect size, stands in need of an explanation. Possible explanations may be found in general differences between x-phi and psychological science, e.g., in terms of differences in publishing cultures, or in the importance of publishing “negative” results. Also differences in experimental design and analysis may also affect the number of reported statistical inconsistencies. After all, one might conjecture that experimental philosophers use straightforward experimental designs and analyses—e.g., yes-no vignettes, small number of variables, t-tests, in comparison to psychologists who may be more likely to use sophisticated statistical techniques—e.g., random effects, generalized linear models, or mediation models.

**Table 3:** Main results of studies investigating the prevalence of statistical reporting inconsistencies in psychology, compared to the current study in experimental philosophy. Table adapted from Table 2 in Nuijten et al. [7]. Percentage of articles with (grossly) inconsistent results computed relative to N = 173.

	No. of Articles downloaded	No. of NHST results (without outlier)	% Inconsis-tencies	% gross inconsis-tencies	% Articles with at least one inconsistency	% Articles with at least one gross inconsistency
Current Study	220	2,558	6.3	0.5	38.7	6.4
[7] Nuijten et al (2016)	30,717	258,105	9.7	1.4	49.6^2^	12.9^2^
[26] Veldkamp et al (2014)	697	8,105	10.6	0.8	63.0	20.5
[27] Bakker and Wicherts (2014)	153^4^	2,667	6.7	1.1	45.1	15.0
[28] Caperos and Pardo (2013)3	186	1,212^3^	12.2	2.3	48.0^2^	17.6^2^
[8] Bakker and Wicherts (2011)	333	4,248^3^	11.9	1.3	45.4	12.4
[6] Wicherts et al. (2011)	49	1,148^1^	4.3	0.9	53.1	

1. Only t, F, and χ2 values with a p < .05

2. Number of articles with at least one (gross) inconsistency/number of articles with null-hypothesis significance testing results

3. Only included t, F, and χ2 values

4. Only articles with at least one completely reported t or F test with a reported p-value < .05

## Discussion

This paper investigated the prevalence of statistical reporting errors in x-phi, using the population of x-phi papers in the PhilPapers database up to September 2016. About 39% of papers that reported NHST results contained at least one inconsistency, and over 6% contained at least one gross inconsistency. Papers presenting gross inconsistencies were characterized by a small systematic bias towards reporting non-significant results as significant, similar to psychological science. One explanation for this finding are *file drawer effects* [29–30]: if significant results have a higher probability to be published, the same holds for gross inconsistencies in the direction of significance. Another explanation is a *double standard in checking results*: experimental philosophers might double-check their analyses more carefully when results are statistically insignificant, than when they are significant. A third possible explanation—which is, however, not supported by the systematic bias we found—appeals to *questionable research practices* (*QRPs*). This would be in line with John et al.’s [5] finding that 22% of the surveyed psychologists admitted to have wrongly rounded down a p-value towards significance. However, our analysis of gross inconsistencies over time did not indicate that QRPs in statistical analysis are on the rise in x-phi, unlike in psychology [31]. With all due caution due to low sample size and selection bias, we also found that differences between subfields of x-phi can be substantial.

Critics have argued that x-phi is just bad psychological science, conducted by researchers without proper training in methodology and statistics (see [32–33] for such objections and [34–35] for responses). According to these critics, x-phi would be in a particularly bad methodological state compared to psychology and other disciplines in the behavioral and social sciences. As far as accuracy of statistical reporting is concerned, however, this hypothesis is contradicted by the results of our study: rates of inconsistencies are lower than for other disciplines in the behavioral and social sciences (39% vs. 45–60%). Leaving subfield differences aside, this suggests that x-phi is not in a worse state than most parts of psychology. That said, one can also speculate about alternative explanations: for instance that x-phi studies use, on average, simpler statistical analysis methods and that this contributes to a lower rate of inconsistent *p*-values.

Our findings cannot answer whether QRPs such as p-hacking and selective reporting are more or less widespread in x-phi than in other behavioral sciences. They fit, however, recent research on the replicability of x-phi findings [25] where x-phi results have been found to be more replicable than experimental studies in psychology.

The reasons for this divergence motivate a number of tentative hypotheses for further research. First, the intensive training in statistical methods that most psychologists and social scientists receive may not be especially effective in preventing inconsistencies in statistical reporting. After all, x-phi achieves lower inconsistency rates without having statistical training as a part of the standard curriculum of a professional philosopher. Second, the abstract reasoning and critical skills that philosophers acquire during their training may help avoiding statistical analyses that are carried out in a mechanical, automatic way [36]. Third, the interdisciplinary nature of most x-phi research may help to avoid reporting inconsistencies by bringing together researchers with diverse training, background and expertise. According to this hypothesis, the lower inconsistency rate would be due to the added methodological value of addressing research questions with collaborators with diverse disciplinary backgrounds and trainings, which is a prominent feature of x-phi research.
